# To Bind or Not to Bind? Different Temporal Binding Effects from Voluntary Pressing and Releasing Actions

**DOI:** 10.1371/journal.pone.0064819

**Published:** 2013-05-31

**Authors:** Ke Zhao, Yu-Hsin Chen, Wen-Jing Yan, Xiaolan Fu

**Affiliations:** State Key Laboratory of Brain and Cognitive Science, Institute of Psychology, Chinese Academy of Sciences, Beijing, China; CSIC-Univ Miguel Hernandez, Spain

## Abstract

Binding effect refers to the perceptual attraction between an action and an outcome leading to a subjective compression of time. Most studies investigating binding effects exclusively employ the “pressing” action without exploring other types of actions. The present study addresses this issue by introducing another action, releasing action or the voluntary lifting of the finger/wrist, to investigate the differences between voluntary pressing and releasing actions. Results reveal that releasing actions led to robust yet short-lived temporal binding effects, whereas pressing condition had steady temporal binding effects up to super-seconds. The two actions also differ in sensitivity to changes in temporal contiguity and contingency, which could be attributed to the difference in awareness of action. Extending upon current models of “willed action,” our results provide insights from a temporal point of view and support the concept of a dual system consisting of predictive motor control and top-down mechanisms.

## Introduction

Subjective compression of the temporal interval between self-generated actions and their consequent outcome demonstrated a perceptual attraction or binding effect towards each other [1]–[4]. Through Libet’s paradigm [5], Haggard et al. set three conditions in which participants performed voluntary key pressing, involuntary (transcranial magnetic stimulation (TMS)-induced) key pressing and sham TMS stimulation [1]. Binding effects occurred in voluntary key pressing condition whereas involuntary movements demonstrated a perceptual repulsion.

What caused this binding effect is debatable though the effect was robust and repeatable. Since binding effect occurred after willed actions [6], one theory attributes distortions of time perception to the speed of the “internal clock” [7]–[9] which varied with stimulation and motor activity [10]. Another theory explains temporal illusions as recalibrations of perceived onset time of sensory events. Predictable events, such as those caused by voluntary actions, may be pre-dated in order to ensure sense of agency [1], [11], causality [12], or perceptual constancy [13]. In both intentional and mechanical conditions, Buehner [14] demonstrated that people will predict target events as occurring earlier if they have a causal understanding of what brings the events about. Thus, suggesting that intentional binding is a specific subset of causal binding and that causality better explained temporal binding. Moore and Haggard [15] argued otherwise and suggest that both intentional actions and causality are equally important. Recent studies by Cravo et al. [16]–[17] also support the idea that both intentionality and causality are necessary by demonstrating that alone, neither intentional actions nor causality leads to binding effects.

The root of temporal binding remains unclear due to discrepancy in time estimation results which led to different findings. It was brought to attention that most investigations based their method on the Libet paradigm, which raises concerns about the external validity of the binding effect. Thus, a novel paradigm employing pushing and pulling actions was proposed and results demonstrated that intentional binding is an externally valid phenomenon [18]. Libet’s experiment (1983) asked participants to perform the quick, abrupt flexion of the finger and/or the wrist of their right hand. This was interpreted by Haggard et al. [1] as a voluntary “pressing” action, whereas Eagleman and Holcombe [2] interpreted it as the voluntary “lifting” of a finger/wrist. Yet, very few studies explored in details whether voluntary action types other than pressing actions could lead to temporal binding. The present study aims to explore this issue by investigating another intentional action, namely releasing action or the voluntary lifting of the finger/wrist, and explore whether it would also lead to temporal binding effects. Several studies [12], [14] suggested that temporal binding is rooted in Hume’s [19] treatise of causation. From Hume’s statement, it can be interpreted that whether a causal relationship forms or not could be determined by the following three factors: *resemblance*, *contiguity*, and *contingency* [19]–[20]. A recent study also revealed that different interstimulus intervals (ISI) and presentation duration of the stimuli also determined whether temporal binding occurred [21]. Extending upon Humphreys and Buehner’s Magnitude Estimation procedure [22], we made a few modifications in order to measure and manipulate factors that were shown to affect temporal binding results, such as temporal contiguity, contingency, presentation duration and ISI. In Experiment 1 we set three conditions: intentional pressing, intentional releasing, and control condition. Participants were asked to estimate the interval between their voluntary action and a visual stimulus. In Experiment 2, we integrated Orgs and Haggard’s (2011) method with Shanks et al.’s paradigm [23] to investigate effects of temporal contiguity through different presentation duration of visual stimulus. In Experiment 3, we devised an experiment that integrated both Experiment 1 and 2, to further explore the effects of temporal contiguity, contingency, and presentation duration of visual stimulus for different ISIs in both intentional pressing and releasing conditions. The purpose of the present study is to explore whether other voluntary actions (e.g. releasing) would lead to different temporal binding results, and if so, investigate the role temporal contiguity, contingency, presentation duration, and ISI plays in creating different temporal binding results. Through these observations we aim to uncover new evidences or details that may help future studies to better determine whether intentional action or causality as the root of temporal binding.

## Experiment 1

### Participants

36 people participated in Experiment 1a (age: 24.17±2.62 yrs; 18 participants; 7 females) and Experiment 1b (age: 22.7±1.84 yrs; 18 participants; 10 females). All participants provided informed consent before the experiment. The experimental procedure was approved by the IRB of the Institute of Psychology, Chinese Academy of Sciences. All participants provided written informed consent before taking part in our experiments. Each participant received 35 Chinese yuan for participating in the 1-hour long experiment. All data from all participants were included.

### Apparatus

A 17-inch cathode-ray tube (CRT) monitor running at a refresh rate of 60 Hz and the software package E-prime 2.0 were used for stimuli presentation and data collection.

### Design and Procedure

There are three conditions in Experiment 1a and 1b: pressing, releasing, and control.

#### Pressing condition

**Figure 1:**
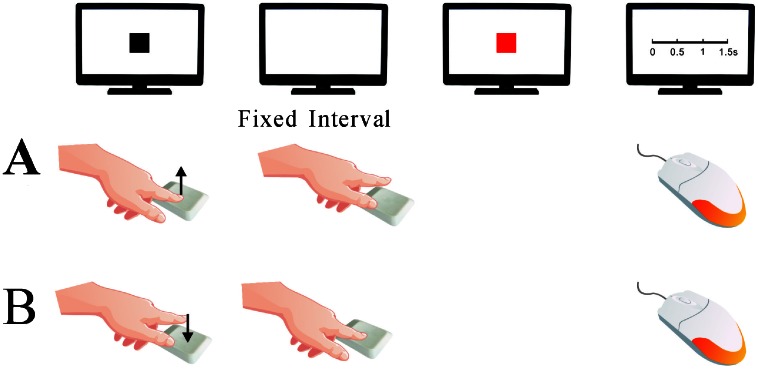
An illustration of a single trial sequence in Experiment 1b for both intentional releasing and pressing condition. A) A black square appears on screen after participants hold down the “F” key, upon releasing the “F” key, the black square would immediately disappear. After a blank screen lasting 450, 650, 850, or 1050 ms, a red square is presented, the subject then completed the time estimation task by clicking anywhere on the time scale. B) An illustration of a trial sequence for intentional pressing condition is depicted.

One black square (size: 1 cm×1 cm) was presented at the center of the screen, participants were asked to place their fingers on the ‘F’ key and press the key when they *intended* to do so. To prevent unconscious pressing and possible mistakes, we set a period of 1.5 seconds as the invalid phase during which the pressing of the ‘F’ key would incur a warning informing participants to restart the present trial. Upon pressing the ‘F’ key after the invalid phase, the black square would disappear immediately. Followed by a blank screen that lasts for a fixed interval (150, 250, 350, or 450 ms for Experiment 1a and 450, 650, 850, or 1050 ms for Experiment 1b) before a red square (size: 1 cm× cm) was presented at the center of the screen for 1000 ms. Participants are then asked to estimate the interval between their *action* and the instant a *red square appears* using the mouse to click on a time scale to provide an answer (ranging from 0 to 600 ms with 5 markers where each division represented 100 ms in Experiment 1a, and a different time scale ranging from 0 to 1.5 seconds with 5 markers where each division represented 0.25 second in Experiment 1b). Participants were able to click on any place on the scale and give a precise number. Then a response-stimulus interval ranging from 1200 to 2000 ms was randomly chosen and presented in between each trial (Figure 1).

#### Releasing condition

A blank screen would be presented indefinitely until participants press and hold down the ‘F’ key which would then present one black square (size: 1 cm×1 cm) at the center of the screen. Participants were asked to release the ‘F’ key when they intended to do so. Upon releasing the ‘F’ key by voluntarily lifting the finger or wrist after an invalid phase (similar to that in pressing condition) the black square would disappear immediately. The rest of the procedure from here on is similar to those in the pressing condition starting from where a blank screen is presented for a fixed interval.

#### Control condition

In this condition participants’ were not required to perform any voluntary action, the black square would disappear automatically after a randomly chosen interval between 1500 to 2500 ms. The rest of the procedure from here on is similar to those in the pressing condition starting from the blank screen presented for a fixed interval.

For Experiment 1a and 1b, each block consists of 28 trials (7 trials for each of the 4 different fixed intervals). Participants performed a total of 3 blocks for each of the 3 conditions (pressing, releasing, and control). Nine blocks were counterbalanced and obtained for each participant for a total of 252 trials each.

Prior to the formal experiment, participants were required to watch 4 examples of different time intervals ranging from 100 to 400 ms to become familiar with the different time intervals in Experiment 1a. In Experiment 1b, participants were required to watch 10 examples of different time intervals ranging from 100 to 1000 ms to become familiar with the different time intervals. Then participants performed 4 trials for each of the 3 conditions to become familiar with the apparatus, procedures, and task.

## Results and Discussion

**Figure 2:**
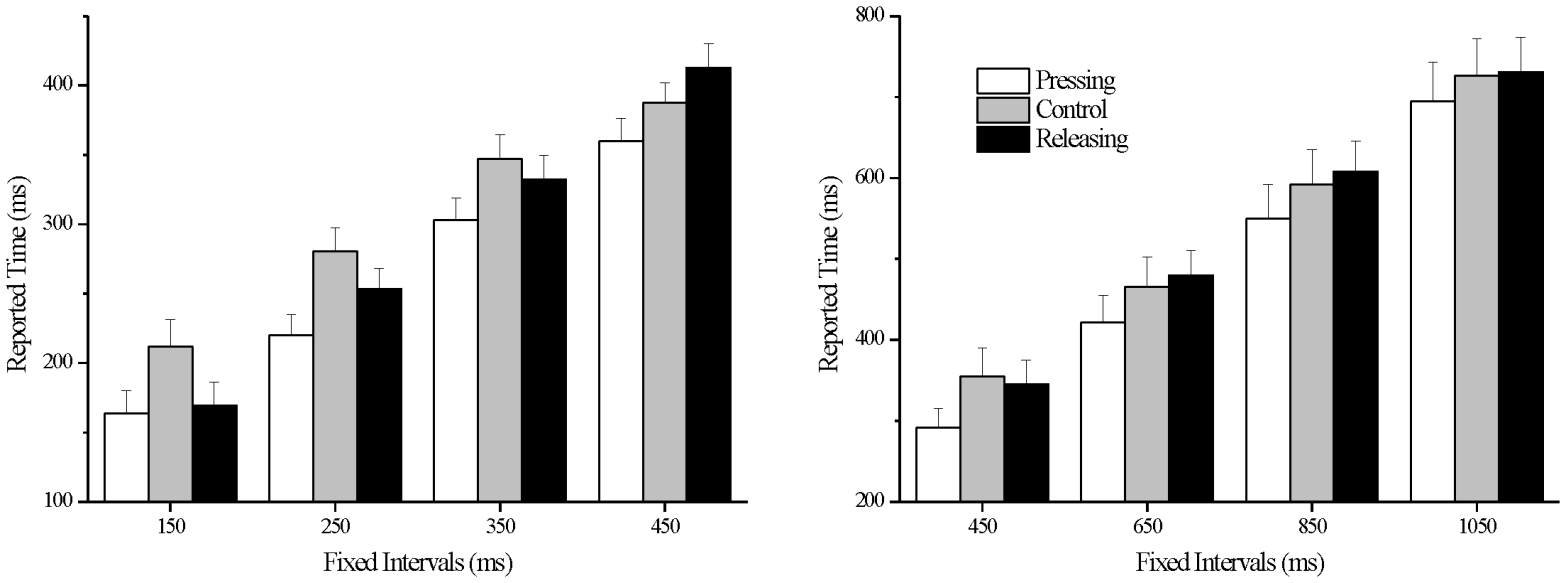
Results for Experiment 1a and Experiment 1b. A represents the subjects’ reported time (y-axis) for the 4 fixed intervals (x-axis) in Experiment 1a. B represents subjects’ reported time for the 4 fixed intervals in Experiment 1b.

A 3 conditions × 4 fixed intervals repeated measures analysis of variance (rANOVA) was conducted on the mean reported time for Experiment 1a and 1b respectively. The main effect of condition was significant, *F*(2, 34) = 8.51, *p* = 0.004, 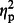 = 0.33. The main effect of fixed interval was significant, *F*(3, 51) = 85.11, *p*<0.001, 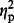 = 0.83. The interaction effect between the two factors was also significant, *F*(6, 102) = 7.77, *p*<0.001, 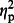 = 0.31. Simple effects analysis revealed that the reported time in the intentional pressing condition and releasing condition were shorter than those of control condition for fixed intervals of 150 ms (*t* = –3.42, *p* = 0.003; *t* = –3.06, *p* = 0.007) and 250 ms (*t* = –3.71, *p* = 0.002; *t* = –1.77, *p* = 0.09). Temporal binding effects were no longer significant in releasing condition for fixed intervals of 350 ms and 450 ms. Whereas, pressing condition had robust and steady effects of temporal binding for all fixed interval in Experiment 1a (Figure 2a).

For Experiment 1b, the main effect of condition was significant, *F*(2, 34) = 4.94, *p* = 0.02, 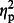 = 0.23. Pairwise comparisons and correction for these comparisons were performed by using the Fisher’s least significant difference. Reported time in the intentional pressing condition (*M* = 489.68 ms, *SE* = 35.53 ms) was shorter than the control condition (*M* = 534.65 ms, *SE* = 37.76 ms) and releasing condition (*M* = 541.22 ms, *SE* = 32.73 ms), *t* = –2.14, *p* = 0.05; *t* = –3.13, *p* = 0.006. The main effect of interval stimulus was significant, *F*(3, 51) = 142.93, *p*<0.001, 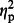 = 0.89 (Figure 2b).

From Experiment 1a and 1b, it can be observed that pressing actions led to steady temporal binding effects up to fixed intervals of 1050 ms. This result is consistent with previous studies that have demonstrated temporal binding effects up to super-second intervals [22]. Interestingly, intentional releasing actions demonstrated robust yet short-lived effects of temporal binding. Results of releasing condition reveal that temporal binding occurred for fixed intervals of 150 and 250 ms only. Reported time for releasing condition not only increased as duration of fixed intervals increased but also matched those of control condition.

Results from Experiment 1 revealed that different voluntary actions lead to different temporal binding results. It has been demonstrated by many studies that causality or predictive motor control models or both are involved in determining binding effect [1], [14]–[17], [22] yet few studies have generated short-lived yet robust temporal binding effect such as those for releasing condition in Experiment 1a. These results probably reflect the additional boost causal binding received from intentional releasing action with consequent outcome following almost *instantaneously*, as was suggested in Buehner’s study [14]. To investigate whether the short-lived temporal binding effect in releasing condition is in anyway related to causality as well as exploring the definition of “almost instantaneous”. We incorporated Orgs and Haggard’s method (2011) for exploring visual stimulus presentation duration with Shanks et al.’s paradigm (1989) to examine the role of temporal contiguity between an action and an outcome in human causality judgments.

## Experiment 2

### Participants

20 people (age: 22.05±2.74 yrs; 7 females) participated in Experiment 2. All participants provided informed consent before the experiment. The experimental procedure was approved by the IRB of the Institute of Psychology, Chinese Academy of Sciences. All participants provided written informed consent before taking part in our experiments. Each participant received 35 Chinese yuan for participating in the 1-hour long experiment. All data from all participants were included.

### Apparatus

A 17-inch cathode-ray tube (CRT) monitor running at a refresh rate of 60 Hz and the software package E-prime 2.0 were used for stimuli presentation and data collection.

### Design and Procedure

There are two conditions for Experiment 2: pressing and releasing. Procedures are similar to those in Experiment 1; participants were asked to hold down or place their finger on the ‘F’ key and wait for 1.5s before releasing or pressing. Upon an intentional pressing/releasing action, however, the black square will be presented for an additional duration of either 0, 30, 60, 90, 120, 150, or 180 ms before it disappears. Followed by the question, “Did you feel a delay in the black square disappearing after you intentionally pressed/released the ‘F’ key?” Participants were required only to answer “Yes” or “No” in this task. If the subject chose “Yes” (feeling a delay of the black square), it is scored “1”; if not, scored “0”. The probability of reported delay was then calculated (i.e. total scores divided by the total trials in each condition).

In Experiment 2, each block consists of 70 trials (10 for each of the 7 presented durations). Participants performed 2 blocks for each of the 2 conditions. The four blocks were counterbalanced, thus each participant performed a total of 280 trials. Prior to the formal experiment, instructions on screen mentioned that a delay may or may not have occurred and it was their task to determine whether a they noticed a delay or not. Participants performed 4 practice trials for each of the two conditions (pressing and releasing) to become familiar with the task as well as experiment procedures.

## Results and Discussion

**Figure 3:**
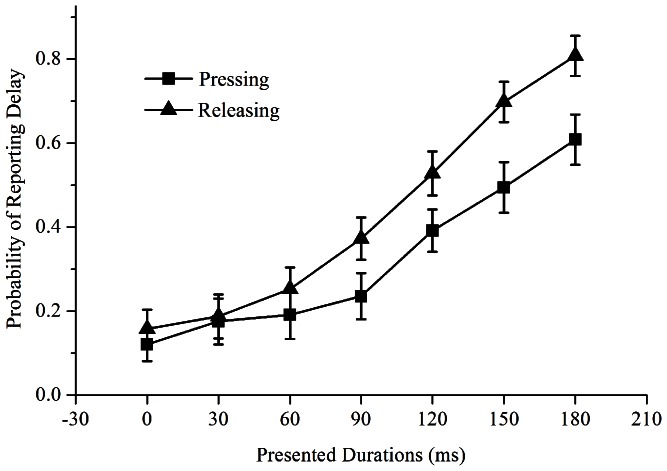
Represents the probability of participants reporting delay for different presented durations (ms).

A 2 conditions × 7 presented durations rANOVA was conducted on probability of reporting delay for Experiment 2. The main effects of presented duration (*F*(6, 114) = 47.59, *p*<0.001, 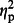 = 0.72) and condition (*F*(1, 19) = 9.29, *p* = 0.007, 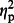 = 0.33) were significant. The interaction between condition and presented duration was also significant *F*(6, 114) = 4.81, *p* = 0.002, 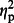 = 0.22. Simple effects analysis indicated that probability of reporting delay in pressing condition was not significant from releasing condition at 0 ms (*t* = –1.02, *p* = 0.32), 30 ms (*t* = –0.25, *p* = 0.80), and 60 ms (*t* = –1.11, *p* = 0.12). However, results revealed a difference between pressing and releasing conditions for presented durations of 90 ms (*t* = –2.18, *p* = 0.04), 120 ms (*t* = –2.72, *p* = 0.01), 150 ms (*t* = –3.87, *p* = 0.001), and 180 ms (*t* = –3.96, *p* = 0.001). From these results, we observed that participants are more likely to report “yes” in the intentional releasing condition than intentional pressing condition for presented durations of 90 ms and longer (Figure 3). As expected, for visual stimulus with presented durations of 180 ms, participants are more likely to notice and report “yes” for both pressing (*M* = 0.61, *SD* = 0.06) and releasing conditions (*M* = 0.81, *SE* = 0.05) revealing that temporal contiguity does affect participants’ judgment of whether an outcome was self-caused or not. Since results from trials with presented durations less than 60 ms were mostly viewed as self-caused. It cannot be determined whether the robust yet short-lived temporal binding effects for releasing condition in Experiment 1a (where no additional presented durations were introduced) occurred was due to an additional boost causal binding received from intentional actions or conscious awareness generated by predictive motor controls [15], [24]. To better understand the results obtained for intentional releasing actions in Experiment 1, we set out to explore how temporal contiguity, contingency, visual stimulus presented duration, and ISI affects participants’ estimation of time for the two action types. In the following experiment, we incorporated the paradigm in Experiment 2 to the paradigm used in Experiment 1 using parameters based on the results obtained in both experiments. In addition, we also developed a questionnaire to explore people’s familiarity of pressing and releasing actions.

## Experiment 3

### Participants

36 people participated in Experiment 3a (age: 23.31±1.78 yrs; 16 participants; 12 females) and Experiment 3b (age: 21.85±1.92 yrs; 20 participants; 13 females). 20 people (age: 23.65±2.76 yrs, 9 females) took our questionnaire. All participants provided informed consent before the experiment. The experimental procedure was approved by the IRB of the Institute of Psychology, Chinese Academy of Sciences. All participants provided written informed consent before taking part in our experiments. All participants performed either the Experiment 3a, 3b, or answered the questionnaire only. Each participant received 35 Chinese yuan for participating in the 1-hour long experiment. All data from all participants were included.

### Apparatus

A 17-inch cathode-ray tube (CRT) monitor running at a refresh rate of 60 Hz and the software package E-prime 2.0 were used for stimuli presentation and data collection.

### Design and Procedure

There are two conditions in Experiment 3: pressing and releasing. The task and procedure were exactly identical to those in Experiment 1, but upon an intentional pressing/releasing action, the black square will be presented for an additional 0, 150, 300, or 450 ms before it disappears. Followed by an ISI of 150, 250, or 350 ms for Experiment 3a and 450, 650, or 850 ms for Experiment 3b before the red square appeared on screen for 1000 ms. Participants were then required to estimate the interval between the black square disappearing and red square appearing on a time scale identical to the one in Experiment 1a and 1b for Experiment 3a and 3b respectively.

For Experiment 3a and 3b, each block consists of 96 trials (8 trials for each of the 4 ISIs and the 3 presented durations). Participants performed 2 blocks for each of the 2 conditions (pressing and releasing). The four blocks were counterbalanced, thus each participant performed a total of 280 trials. Training procedures identical to those in Experiment 1 were performed for participants to become familiar to the different sub-second time intervals. Then participants performed 4 practice trials each for pressing and releasing conditions.

A questionnaire was designed to survey people’s familiarity of the pressing and releasing actions. Participants had to write as many items that could be manipulated/activated/controlled in their daily life through pressing or releasing action given one minute per question. Results showed that items written for pressing (*M* = 4.05, *SD* = 1.23) were more than those for releasing (*M* = 1.15, *SD* = 0.98), *t* = 8.96, *p*<0.001. Afterwards, a 7-point familiarity scale (“1” representing the least familiar and “7” representing the most familiar) indicated that participants were more familiar with pressing action (*M* = 6.45, *SD* = 0.68) compared to releasing action (*M* = 2.95, *SD* = 1.70), *t* = 9.75, *p*<0.001.

### Results and Discussion

Analyses of variance were conducted on participants’ relative reported time to ensure normality. A 2 conditions×3 ISIs×4 presented durations rANOVA was conducted on the mean relative reported time for Experiment 3a and 3b respectively. Relative reported time for each trial was obtained by subtracting actual estimated time by the fixed interval of the given trial then divided by the fixed interval of the given trial.

**Figure 4:**
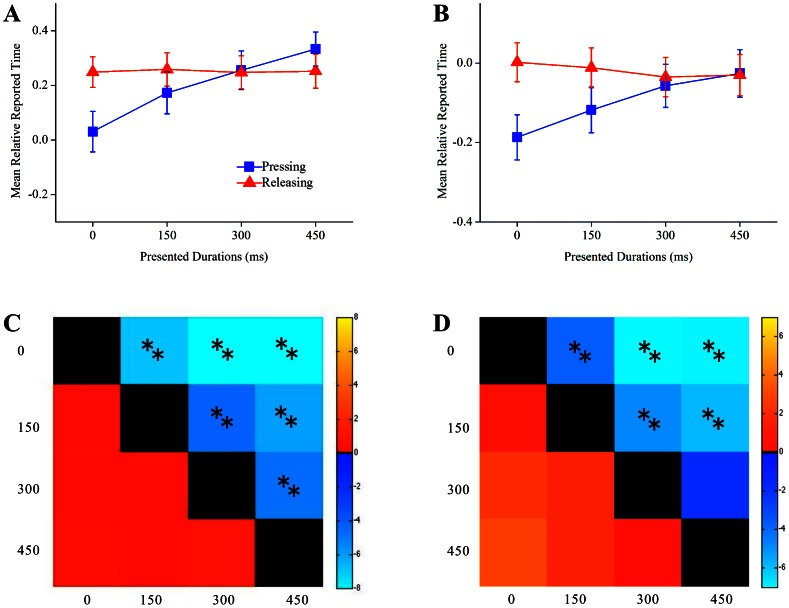
Results of experiment 3a and experiment 3b are shown in A, C and B, D respectively. A and B represent participants’ mean relative reported time for each presentation duration in both pressing and releasing conditions. C and D represent a comparison where the brighter the color the larger the difference of mean relative reported time is between any two presented durations. The color blue is used to indicate mean relative reported time (t-value) between two presented durations in pressing condition. While the color red is used to indicate mean relative reported time (t-value) between two presented durations in releasing condition. Two stars indicate that p<0.01 and one star indicates that p<0.05. Black color represents zero difference.

For Experiment 3a, the main effects of presented duration (*F*(3, 45) = 37.39, *p*<0.001, 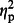 = 0.71), ISI (*F*(2, 30) = 9.57, *p* = 0.007, 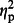 = 0.39), and condition were significant (*F*(1, 15) = 4.31, *p* = 0.055, 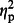 = 0.223). The interaction between condition and presented duration was significant (*F*(3, 45) = 37.79, *p*<0.001, 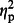 = 0.72). Simple effects analysis indicated that mean relative reported time was shorter in pressing condition than releasing condition for presented durations of 0 ms (*t* = –6.32, *p*<0.001) and 150 ms (*t* = –2.29, *p* = 0.037). However the mean relative reported time showed no difference when presented duration increased to 300 ms (*t* = 0.28, *p* = 0.78). The mean relative reported time was longer in pressing condition than releasing condition for the duration of 450 ms (*t* = 3.11, *p* = 0.007). No difference were found in the mean relative reported time for releasing condition given any two presented durations, whilst mean relative reported time displayed differences given any two presented durations for pressing condition (Figure 4a and 4c).

For Experiment 3b, the main effects of presented duration (*F*(3, 57) = 11.78, *p*<0.001, 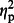 = 0.38), ISI (*F*(2, 38) = 0.24, *p* = 0.643, 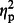 = 0.012), and condition were significant (*F*(1, 19) = 8.37, *p* = 0.009, 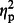 = 0.30). The interaction between condition and presented duration was significant (*F*(3, 57) = 28.43, *p*<0.001, 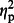 = 0.59). Simple effects analysis indicated that mean relative reported time was shorter in pressing condition than releasing condition for presented durations of 0 ms (*t* = –5.30, *p*<0.001) and 150 ms (*t* = –3.68, *p* = 0.002). However the mean relative reported time showed no difference when presented duration increased to 300 ms (*t* = –0.89, *p* = 0.39) and 450 ms (*t* = 0.13, *p* = 0.90). Relationship between mean relative reported time and presented duration was similar to those in Experiment 3a, for more details see Figure 4b and 4d.

Results from Experiment 3 demonstrated how modulations to the temporal contiguity of an action and outcome determined whether temporal binding occurred or not. In trials where no additional presented duration were introduced, participants’ reported time in the pressing condition was significantly shorter than those compared to releasing condition. However, as presented duration lengthened to 300 or 450 ms, a concomitant decrement in temporal binding occurred just as previous studies have suggested [25] (Figure 4a and 4b). Oddly, as presented duration increased the causal efficacy of releasing action and the outcome did not lead to a concomitant decrement in temporal binding as those seen in pressing condition. Results of releasing condition revealed that the different presented durations did not affect participants’ judgment in time estimation in low contingent trials (Figure 4c and 4d).

Reported time for both pressing and releasing conditions with identical ISI (150, 250, and 350 ms) and no additional presented duration in Experiment 3 were longer than those of Experiment 1 as expected due to a reduction in contingency (Table 1). In Experiment 1a, since *all* intentional releasing actions would lead to the immediate disappearance of the black square. The probability of an action leading to an instantaneous disappearance of the black square would be 1.00, whereas in Experiment 3a, there is only a probability of 0.25 (0.75 chance that an additional presented duration of 150, 300, or 450 ms is introduced) that the action would lead to the instantaneous disappearance of the black square. It could be inferred that the change in contingency in Experiment 3 led to a reduction in causal efficacy between action and outcome which may have negated the additional boost causal binding recieves from intentional action. Similarly, it could also be interpreted that recent prior experience on predictive motor control abolished the predictive binding effect [15].

**Table 1:** Mean reported time for different fixed intervals between experiment 1a and experiment 3a are listed.

		150	250	350
**Experiment 1a**	Pressing	163.81(16.29)	220.05(14.79)	303.32(15.79)
	Control	211.71(19.94)	280.29(16.92)	347.23(17.14)
	Releasing	169.31(17.11)	253.42(14.56)	332.36(17.24)
**Experiment 3a**	Pressing	184.24(19.93)	249.92(18.22)	302.57(15.45)
	Releasing	218.58(18.42)	294.47(11.71)	389.03(15.33)

Mean reported time for Experiment 3a listed in this table only includes data where no additional presentation duration (0 ms) were introduced to observe the effects of low contingency (0.25) in Experiment 3a to high contingency (1.00) in Experiment 1a. Numbers in parenthesis represent the standard deviation of mean reported time.

## General Discussion

Our results demonstrated how changes in contingency and temporal contiguity via modulation of visual stimulus presented duration and different ISI plays a critical role in determining whether temporal binding occurs or not. In Experiment 3, pressing condition showed robust results that demonstrated temporal binding effects up to ISI of 850 ms when no additional presented durations were injected in spite of low contingency. Pressing actions also revealed robust temporal binding effects for trials with presented durations of 150 ms in Experiment 3. However, as presented duration between action and the disappearance of the black square increased to 300 and 450 ms, a concomitant decrement in temporal binding occurred as expected [25].

In contrast, the reported time for releasing condition was more sensitive to changes in temporal contiguity and contingency. In fact, trials in Experiment 3a with ISI of 150 ms and no additional presented durations injected did not lead to the same short-lived temporal binding effects that were observed in Experiment 1a (Table 1) possibly due to changes in contingency. This result is consistent with several studies which demonstrated that both intentional action and high contingency is required to lead to binding effects [15]–[16].

According to these results, the robust yet short-lived temporal binding effects of releasing condition in Experiment 1a could be that causal binding receives an additional boost from intentional action if the consequence follows almost instantaneously [14]. However, causality alone is incapable of explaining both the results of pressing as well as releasing conditions. Take the case of intentional releasing, if we were to explain the results of Experiment 1 through causal binding, one would explain that stronger causal relations exist for fixed intervals of 150 and 250 ms due to stronger temporal contiguity than fixed intervals of 350 and 450 ms. Therefore, it follows that a robust temporal binding effect in fixed intervals of 150 and 250 ms occurred, whereas fixed intervals of 350 ms and above are too far apart in time to have strong causal relations thus no temporal binding effect occurred. Unfortunately, if this observation was true, it would contradict with results in pressing condition that revealed steady temporal binding effects up to fixed intervals of 1050 ms. Vice versa, if we were to explain temporal binding results of pressing condition through causal binding, the explanation would also be contradictive with results of releasing condition. Similarly, intentional binding faces the same problem as causal binding when faced with explaining the different temporal binding results of intentional pressing and releasing actions in Experiment 1. If binding effect is rooted in voluntary actions, then intentional releasing action should lead to similar temporal binding effects that last up to super-seconds like those demonstrated in pressing condition.

From our results, we observed that pressing actions are less sensitive to changes made towards factors of causality (temporal contiguity and contingency). It is likely that the two different action types procured two temporal binding results with such striking difference due to different levels of action awareness, in other words, sense of agency. From results of our questionnaire, we observe that participants are more familiar with pressing actions in comparison to releasing actions. This is consistent with results in Experiment 2 where participants were more “blind” to the presented duration we introduced in trials that employ the pressing action. This stronger feeling of agency in pressing action is likely to be a result of contingency learning [12]. Since, people generally operate a lot of machineries through pressing action; people are more tolerant of the changes in temporal contiguity and contingency when performing pressing actions because experience tells them that “pressing action” *tends* to generate a certain outcome and through contingency learning, such a causal relation has been established and strengthened. Thus, even in low contingent trials and weak temporal contiguity, temporal binding effects were still visible in Experiment 3 for pressing condition. Releasing actions however are less well-known, from the results of our questionnaire we can observe that participants are not able to list as many machineries or outcomes that are generated or related to releasing action. Lower awareness to releasing action may have caused participants to reject the notion that releasing action is capable of leading to an outcome. Therefore, aside from short-lived temporal binding results in Experiment 1a, releasing actions did not lead to temporal binding effects in any other conditions and experiments. As for the short-lived temporal binding result that occurred in Experiment 1a, it is possible that we obtained results similar to Moore and Haggard’s (2008) findings that suggest recent prior experience influenced the predictive contribution to action awareness. Such that action awareness reflects short-term learning and in part, subserves predictions made by the motor control system. Yet, it seems odd that such temporal binding effects obtained from releasing action only lasted up to 250 ms in Experiment 1a.

It has been suggested that both intentional action and causality is necessary for temporal binding effect to occur [15]–[16] and several studies have established models to explain how predictive and inferential processes are carried out to conduct a “willed action” [26]–[27]. Our results also seem to support current models and may provide additional insights from a temporal point of view. Lewis and Miall [28] proposed that time measurements of sub-second durations or durations defined by movement may involve motor circuitry. Studies have also suggested that time measurements in the sub-seconds range are automatic, whereas measurements in the multi-second range require attention [29]–[30]. Regarding top-down cognitive processes, time (or lack thereof) is suggested to act as a gatekeeper that determines whether cognitive control processes can unfold [31]. Results from Experiment 1 seem to support these findings about time and the notion of a dual system for “willed actions” would better explain the robust yet short-lived temporal binding results of releasing actions. Our results suggest that under ISIs of 250 ms, “willed actions” are predominantly affected by information from the motor control system. Whereas willed actions that involve ISIs of 350 ms and higher are domineered by retrospective inferential processes such as causality and sense of agency. Similar observations were also seen in another study exploring temporal binding mediated by a top-down mechanism [21]. In their study, results revealed that plausible biological motion perception were more likely to be perceived by participants for ISIs of 300 ms, since longer duration increases available processing time. Alike the two systems of reasoning proposed by Sloman [32], our results suggest that predictive motor control systems generally determine actions and their respective consequences under 250 ms. Top-down mechanisms, cognitive processes, and causality can override motor control system’s judgments and predictions if the brain is provided with sufficient processing time [33]–[34].

The present study aimed to explore whether different temporal binding results would occur when different voluntary actions were performed. Results revealed that the two different action types explored in our study procured two different temporal binding results. Further investigation revealed that pressing and releasing actions are different in two aspects: 1) the two actions differ in sensitivity to changes in factors of causality such as temporal contiguity and contingency. 2) difference may also be attributed to action awareness with pressing action possessing stronger awareness due to constant contingency learning, whereas participants are less aware of releasing actions. Our findings support the concept of a dual system for “willed actions” where predictive motor control generally determines actions and their outcomes for ISIs less than 250 ms. Whereas top-down mechanisms (i.e. sense of agency and causality) not only determines but also influences subjective perception of action and their outcomes if provided with sufficient processing time.
